# Impact of Structural Functionalization, Pore Size, and Presence of Extra-Framework Ions on the Capture of Gaseous I_2_ by MOF Materials

**DOI:** 10.3390/nano11092245

**Published:** 2021-08-30

**Authors:** Fabrice Salles, Jerzy Zajac

**Affiliations:** ICGM, Université Montpellier CNRS ENSCM, Montpellier, France; jerzy.zajac@umontpellier.fr

**Keywords:** Monte Carlo simulations, adsorption, Metal–Organic Framework, gaseous iodine, fission gases

## Abstract

A computational approach is used on MOF materials to predict the structures showing the best performances for I_2_ adsorption as a function of the functionalization, the pore size, the presence of the compensating ions, and the flexibility on which to base future improvements in selected materials in view of their targeted application. Such an approach can be generalized for the adsorption of other gases or vapors. Following the results from the simulations, it was evidenced that the maximum capacity of I_2_ adsorption by MOF solids with longer organic moieties and larger pores could exceed that of previously tested materials. In particular, the best retention performance was evidenced for MIL-100-BTB. However, if the capacity to retain traces of gaseous I_2_ on the surface is considered, MIL-101-2CH_3_, MIL-101-2CF_3_, and UiO-66-2CH_3_ appear more promising. Furthermore, the impact of temperature is also investigated.

## 1. Introduction

The energy production in nuclear power plants during normal operating conditions induces greenhouse gas emissions, which are lower than those generated by fossil fuel technologies or, at the most, comparable to those accompanying electricity production based on renewable energy sources [1]. A more serious problem related to safe nuclear power concerns the potential radiological release of fission gases that are formed during fuel reprocessing or nuclear fallout [2]. Among others, the capture of gaseous I_2_ to avoid its easy spread into the environment remains a real challenge. The impact of this element on the human health can be harmful for long periods since, e.g., ^129^I has a half-life of 15.7 million years. Furthermore, the high volatility of iodine and its eventual involvement in human metabolic processes, representing an immediate threat for the population, have inspired the quick development of a variety of removal technologies [3].

The selective removal of iodine directly from the gaseous phase by adsorption onto porous materials, thereby limiting the presence of its radioactive vapors in atmosphere, may be a good alternative to the more classical absorption of gaseous constituents by scrubbing into a liquid solvent [4]. The main research strategy here is to propose solid adsorbents that exhibit a strong affinity towards iodine at low surface coverages (valuable solution in the case of I_2_ traces in the off-gas) or adsorbents offering a high retention capacity necessary to store I_2_ molecules in large quantities. Solid materials that have already been tested for such uses include zeolites [4], mesoporous silicas functionalized with silver nanoparticles [5], and clathrates [6]. The strong binding affinity of gaseous iodine units for metal surface sites has motivated research interest in metal–organic frameworks (MOFs) that have proven to be highly efficient in various applications where sorption phenomena underlie retention mechanisms [7,8,9,10,11,12]. These materials are composed of metal centers organized in chains or clusters and linked to one another through organic linkers. Due to their high structural and chemical versatility, they have shown remarkable performances when capturing gas components such as H_2_, CO_2_, CH_4_, and their mixtures [13,14,15,16]. Other applications such as catalysis, medical technology, energy storage, and conversion also deserve to be mentioned, especially if the use of composites is accompanied by a special effort to propose green synthesis to reduce environmental impacts during their preparation [17,18,19,20]. The surface activity of MOFs upon adsorption and separation of gases can be altered through different approaches, as has been already reported in the literature [21,22,23,24,25]. For example, it was possible to carry out functionalization of the organic part and/or modification of the metal center in order to tune interactions occurring between the adsorbate and the solid surface, thereby modelling the adsorption power of the adsorbent [26,27,28,29,30,31,32] and its flexibility capacity [33,34]. Furthermore, the usefulness of these porous solids for nuclear applications has been already publicized, since some MOF materials are considered for the adsorption of a radioactive gas [35,36,37,38,39,40] or ion sequestration [41,42].

From an experimental viewpoint, correctly assessing the real decontamination performance of adsorbents is not an easy task because of the potential health risks following exposure to radiation. Moreover, this task may appear fastidious if one takes into account the large number of various MOF samples that can be achieved and tested. In such circumstances, molecular simulation methods offer a powerful and fast tool to evaluate the sorption capacity of different hybrid solids on which to base their subsequent optimization and the selection of the best candidate for their targeted uses. Such a strategy has already been followed when studying the adsorption of H_2_, CO_2_, and other gases [25,43,44,45]. By far the greatest added value provided by molecular simulations is the possibility of describing, at a microscopic level, the mechanisms involved [46,47,48,49,50,51].

Concerning the retention of I_2_ from the gas phase, ZIF-8 is the most frequently investigated adsorbent of the MOF family [52,53]. Strong interactions between the I_2_ molecules and the imidazolate moieties were indicated to account for an adsorbate retention four times higher than that of activated carbons [53]. Considering various MOF materials with aluminum as a metal center, Loiseau et al. showed that the sorption of I_2_ in combination with liquid cyclohexane was the highest on samples containing a grafted NH_2_ group when compared to pristine materials [54]. On the other hand, computational studies have also been performed to screen the performances of other selected MOFs [55,56,57]. Furthermore, some sorbents for radionuclide species have also been designed from UiO-66 and ZIFs series with enhanced retention performances [58].

The intention of the present paper is to consider the impact of: (i) structure functionalization, (ii) the size of the pores, and (iii) the presence of extra-framework counter-ions on both the adsorption performance of selected MOF materials towards gaseous I_2_ and their adsorption mechanisms. The starting MOF materials included a microporous MIL-53 series [33], a mesoporous MIL-100 and MIL-101 series [59], MIL-127 [60], Zn-BTeC [61], and a UiO-66 series [62]. The different structures investigated are shown together in the Materials and Methods section. As it has been shown that the capture of radioactive contaminants using dry processes (namely, using porous solids without trace of water) can be an important solution for industry [63], we mainly focus here on the adsorption of pure I_2_ in porous solids.

## 2. Materials and Methods

Prior to our calculation of the adsorption isotherms, our main effort was focused on determining the structure of each MOF solid considered in this work. For this purpose, we considered the following structures already published in the literature: (a) the MIL-53 series bearing a H, 1CH_3_, NH_2_, Br, or Cl functional group grafted on the phenyl ring of benzenedicarboxylate linker (bdc)) [33]; (b) an MIL-100 series possessing a bdc or 1,3,5-tris(4-carboxyphenyl)benzene (BTB) group as an organic moiety) [59]; (c) an MIL-101 series with a H, NH_2_, Br, CF_3_, Cl, or 2CH_3_ group on the phenyl ring of a bdc linker, or those containing a 2,6-naphthalenedicarboxylate (NDC), 4,4′-biphenyldicarboxylate (BPDC) group as an organic moiety) [59]; (d) a UiO-66 series with a H, Br, CH_3_, Cl, NH_2_, NO_2_, or 2CH_3_ group grafted on the phenyl ring of a bdc part [62].

Concerning the MIL-53 series investigated here, the ‘open’ forms (or large-pore forms) of these structures were considered to determine the maximum amount of I_2_ adsorbed. In this particular case, the experimental parameters of the unit cell were fully imposed.

All structural models were then energy-minimized within the P1 space group by keeping the cell parameters fixed, using the universal force field (UFF) and charges calculated from the qEq method, as implemented in Materials Studio software [64]. Such a strategy has already been successfully employed to construct plausible structures of various MOFs [33,59,65]. The Ewald summation was considered for calculating electrostatic interactions while short-range interactions were evaluated using a cut-off distance of 12 Å. The convergence criteria were set at: 1.0 × 10^−4^ kcal mol^−1^ (energy), 0.005 kcal mol^−1^ Å^−1^ (forces), and 5.0 × 10^−5^ Å (displacement). All geometry optimizations converged to provide a plausible crystallographic structure for each MOF investigated in this paper.

A classical molecular simulation was subsequently carried out to calculate both the adsorption isotherm for I_2_ and the adsorption enthalpy at low coverages. Grand Canonical Monte Carlo (GCMC) simulations were performed making use of the SORPTION (from Materials Studio) or home-made code, typically with 2.0 × 10^6^ Monte Carlo steps for production, followed by 5 × 10^6^ steps for equilibration [66]. The Ewald summation was also used for calculating electrostatic interactions while short-range contributions were computed with a cut-off distance of 12 Å. The simulations were conducted at 300 K using the previously simulated structures considered as rigid and with cell parameters allowing for the use of a 12 Å cut-off distance. Again, a UFF force field was used in regard to the framework atoms and I_2_ molecules, as proposed by Nenoff et al., who developed a diatomic model for I_2_ without explicitly considering polarizability [53]. Our interest in this force field was to consider that the electrostatic part of the energy had only a small influence, and that a van der Waals interactions played a main role in the adsorption phenomenon, as already proven in the case of various porous solids [67,68,69,70]. It can also be noted that the force field developed by Nenoff was able to reproduce the interactions of I_2_ with Ag^+^-mordenite, even without taking into account electrostatic interactions. This justifies the use of this force field to investigate the selected solids in the present case. Furthermore, DFT calculations provided strong indications that the van der Waals forces played a predominant role in the interactions between I_2_ and MOF, thereby suggesting that the electrostatic effect may be neglected as a first approximation [69].

A similar calculation strategy was repeated at 353 K so as to verify whether the observed trends would be transposable to other conditions of temperature reported in previously published experimental studies.

From the Monte Carlo simulations, it was possible to extract the most plausible configurations that corresponded to the most probable distribution of the guest I_2_ molecules inside the pores of the different solids investigated within the framework of the present study. The distances reported in the snapshots included in the manuscript are consistent with distances obtained from radial distribution functions. They allow us to elucidate typical interactions and, therefore, to clarify the main interaction sites for I_2_ adsorption.

Using these structures, textural properties (specific surface area, SSA, and pore volume, PV) were calculated by considering the strategy previously developed by Düren et al. [71]. From the area defined by the motion of the center of a nitrogen molecule rolling along the surface, it was possible to calculate SSA values. The diameter of the probe molecule was considered to be equal to 3.681 Å, whereas the diameter of each atom constituting the MOF structure was taken from the UFF force field data [64]. The PV was calculated for each simulated structure using a similar method of trial insertions within the entire volume of the unit cell. A 0 Å-size probe was used for the determination of the free volume of the unit cell unoccupied by framework atoms [71].

## 3. Results and Discussion

**Figure 1:**
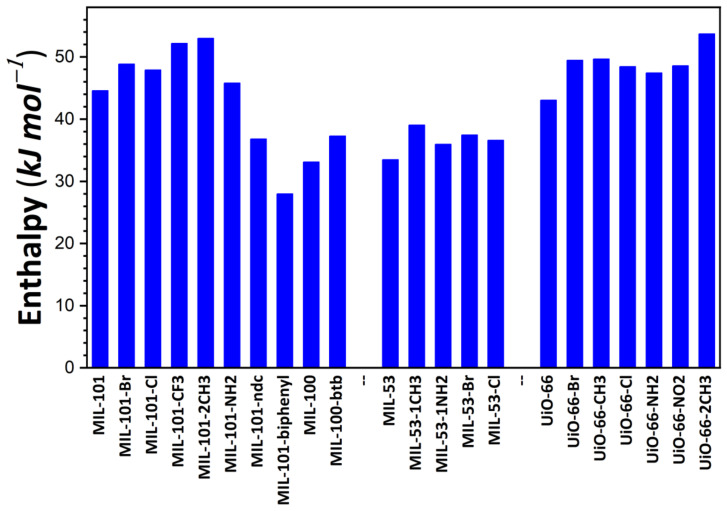
Energy release during the adsorption of the first I_2_ molecule onto MOF structures studied in the present paper, as calculated based on the Monte Carlo simulations.

The energy released during the adsorption of the first I_2_ molecule within the pore space was taken as the first criterion to estimate the performance of the adsorbents. This particular energy value was considered as the measure of adsorbate–adsorbent interaction strength in the presence of traces of I_2_ vapor. Figure 1 illustrates the variations in this energy as a function of the chemical and structural features of the studied materials. Concerning the MIL-100/MIL-101 family, energy values range between 28 kJ·mol^−1^ for MIL-101-biphenyl and 52 kJ·mol^−1^ for MIL-101-2CH_3_. Such a variation can mainly be explained by the introduction of chemical functionalization (organic moieties). It is likely induced by a modification of pore volume due to the confinement effect as a consequence of the introduction of various chemical moieties. A comparison with the energy variation at low surface coverages recorded on samples belonging to the MIL-53 family clearly shows that the effect of functionalization is drastically decreased when one considers the solid structures with lower pore volumes. This can be explained by the fact that the modification of pore volume through the introduction of chemical functions is strongly limited. Indeed, the MIL-100/MIL-101 structures have larger pores than those of the MIL-53 series. It is worth noting that, in the present calculations, the MIL-53 series was taken in an ‘open’, large-pore form. For such structures, energy changes between 34 kJ·mol^−1^ for MIL-53 and 48 kJ·mol^−1^ for MIL-53-1CH_3_ were observed, in good agreement with the experimental trend reported in [54]. In the case of the UiO-66 series, an intermediary effect was recorded for MIL-53 and the MIL-100/MIL-101 series, with energy values of 42 kJ·mol^−1^ for UiO-66 and 53 kJ·mol^−1^ for UiO-66-2CH_3_. This description may be completed by taking into account the observation that MIL-127 saturated with Cl^−^, I^−^, or NO_3_^−^ releases the same amounts of energy (i.e., close to 35 kJ·mol^−1^), while the energy values obtained with Zn-BTeC saturated with alkali cations (Li^+^ to Cs^+^) range from 41 to 45 kJ·mol^−1^. All values reported here are higher than the vaporization enthalpy of I_2_ at ambient temperature, which is close to 20 kJ·mol^−1^. Given these results of energy calculation, MOFs characterized by a neutral framework appear effective in capturing traces of gaseous I_2_ released upon nuclear and radiation accidents and incidents; however, ion-containing structures do not involve strong interactions with this vapor, but only relatively strong interactions that are weakly influenced by the nature of the compensating ions.

A comparison of the present results with those reported on the basis of other computational studies shows a good agreement with other classical simulations. Indeed Assfour et al. [55] evaluated isosteric heat using different adsorption isotherms calculated at different temperatures and found 30 kJ·mol^−1^ for MIL-100 and 55 kJ·mol^−1^ for MIL-53(Al).

To better evaluate the impact of structural functionalization, the interactions between the adsorbate and the adsorbent were investigated by considering the plausible molecular configurations within the pores for each sample. It should be emphasized that plausible molecular configurations correspond to the distribution of guest I_2_ molecules in porous solids as a function of the statistical distribution extracted from Monte Carlo simulations. The related snapshots are reported in the Supplementary Materials. In the case of the MIL-53 series, the observed impact of the functional groups grafted on the organic moieties is rather weak. Indeed, all distances reported in Figure S1 are more or less identical. Only small changes can be noted for the NH_2_ group. The same behavior can be observed for the MIL-101 series (Figure S2), where the NH_2_ function likely interacts with the I_2_ molecule. However, the effect is only slightly noticeable, which is probably due to the fact that pore sizes are relatively large in line with the open form of the MIL-53 solids. On the contrary, the samples belonging to the UiO-66 series possess smaller pores, and stronger interactions are detected at smaller distances between I_2_ and the organic linker (Figure S3). This means that, even for the first adsorbing molecules, the nature of the ligand has an effect only when small pores are considered. It is also important to note at this level that the choice of the force field implemented in the calculation code may have some impact on the results obtained. Electrostatic contributions to the adsorbate–adsorbent interactions were neglected here. The development of a force field including such a contribution may certainly be of interest to elucidate the full impact of structural functionalization. However, to date, no calorimetric data are available in the literature, thereby leaving such a force field essentially untested. Based on the present results of our calculations, it is possible to hypothesize that the NH_2_ functional group is promising in view of use of MOF materials in nuclear safety applications.

The affinity of the solid surface towards I_2_ and the saturation plateau are two other parameters to investigate. To determine them, the I_2_ adsorption isotherms were calculated for each solid sample; the results are reported in the Supplementary Materials (Figures S4–S9). The affinity parameter corresponds to the slope of the isotherm at low pressures. A comparison between all solids clearly indicates that the MIL-53 and UiO-66 series present a strong affinity, whereas the MIL-100/MIL-101 ones are characterized by a drastically lower affinity towards I_2_. This result is thus in good agreement with the conclusions presented before with respect to the adsorption enthalpy calculated at low I_2_ loadings.

If we consider the saturation plateau for each isotherm, some trends may be inferred from the results of our calculations. In the case of MIL-53, UiO-66, and the MIL-101 series, the MOF structures containing H or NH_2_ groups appear the most performant in adsorbing large quantities of I_2_. The order of increasing I_2_ adsorption capacity was as follows: Cl ≈ NH_2_ ≈ H > CH_3_ > Br, MIL-53 series (in agreement with trends obtained from experimental data in the liquid phase [54]); NH_2_ ≈ H > 2CH_3_ > Cl > Br > CF_3_, MIL-101 series; H > NH_2_ > CH_3_ > Cl > 2CH_3_ > NO_2_ > Br, UiO-66 series. A comparison of these theoretical values with the experimental results shows a relative good agreement, as UiO-66 saturation reaches 0.7 g·g^−1^ [70]. The difference can be explained by the accessibility of the entire porosity achieved in the theoretical model in contrast with the experiment. The maximum amount of I_2_ adsorbed was reported to be about 4 g·g^−1^ in the cases of MIL-101-NH_2_ and MIL-101-H (c.f., Figure S3). However, grafting a longer organic linker should result in a much higher adsorption capacity, e.g., the 10 g·g^−1^ for MIL-100-BTB at ambient temperature. In contrast, much lower adsorption capacities were obtained with ionic MOFs (i.e., Zn-BTeC and MIL-127), and they were found to be only slightly dependent on the nature of the compensating ion present in the pores. Such predicted values are still higher than the best reports, such as in Zr-based MOF-808 at 80 °C (2.18 g·g^−1^) without water [70], or MOF-808 covered by poly(vinylidene fluoride) (1.42 g·g^−1^) [72]. It should be noted that a comparison with experimental data is difficult due to the scarcity of the experimental reports. Taking as an example the reference [54], the saturation measured in MIL-53(Al)-NH_2_, MIL-100, and MIL-101 at room temperature (respectively 150, 50 and 350 mg·g^−1^) is much lower than the theoretical saturations obtained in the present study. However, such experimental values are not consistent with the pore volume (respectively, 0.56, 1 and 1.8 cm^3^·g^−1^), thereby suggesting that other phenomena occur upon I_2_ adsorption (adsorption of other molecules, swelling or degradation of the solid under the conditions applied, or others). Other theoretical and experimental studies are more in line with our simulations, as the saturation of MIL-53(Al) is close to 1 g·g^−1^, MIL-101 reaches 5 g·g^−1^ [55], while UiO-66 can adsorb 1.17 g·g^−1^.

**Figure 2:**
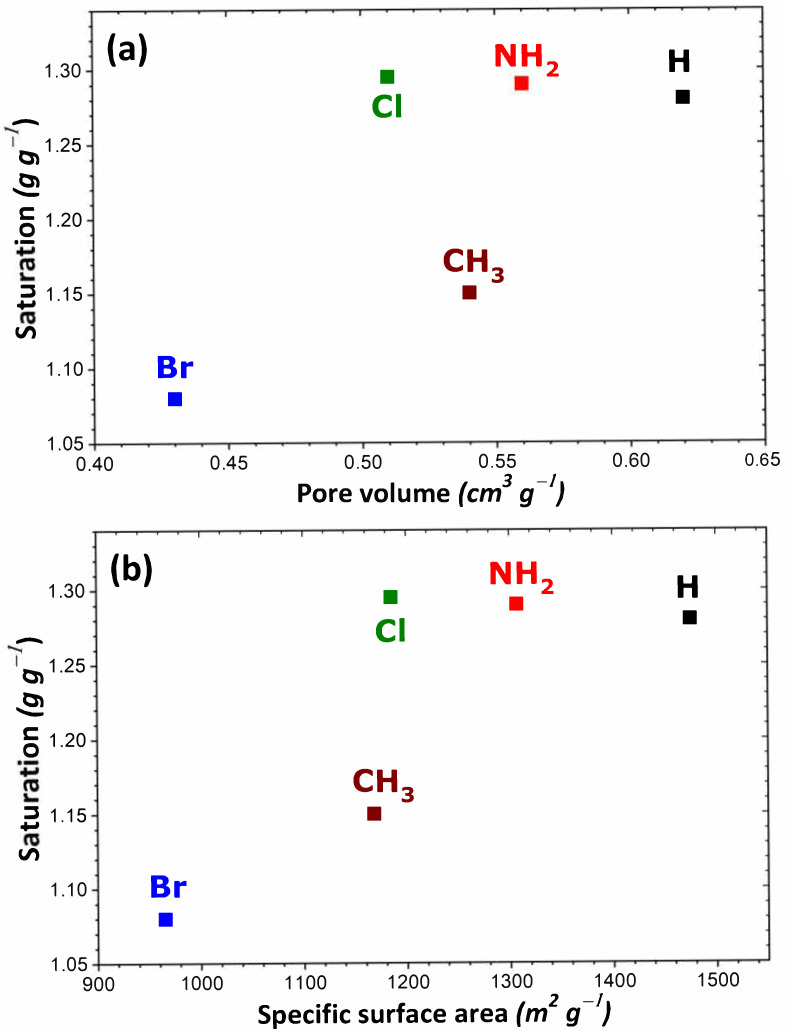
Evolution of the adsorption capacity of the adsorbents belonging to the MIL-53 series towards gaseous I_2_ at saturation as a function of pore volume (**a**) and specific surface area (**b**).

**Figure 3:**
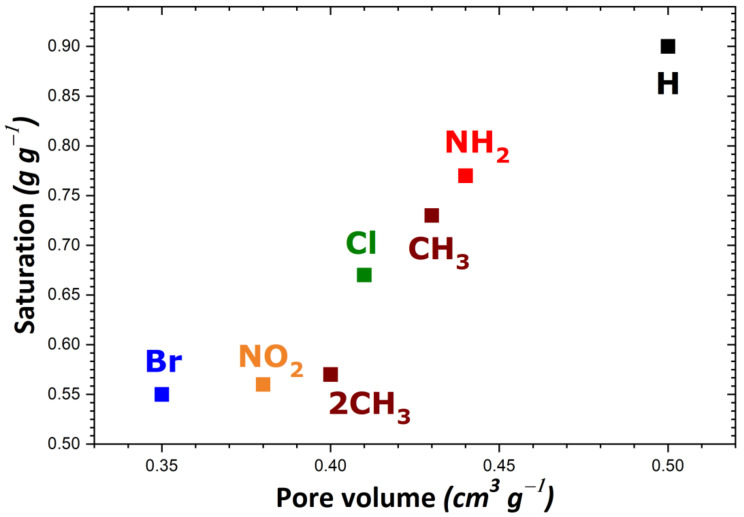
Evolution of the adsorption capacity of the adsorbents belonging to the UiO-66 series towards gaseous I_2_ at saturation as a function of pore volume.

**Figure 4:**
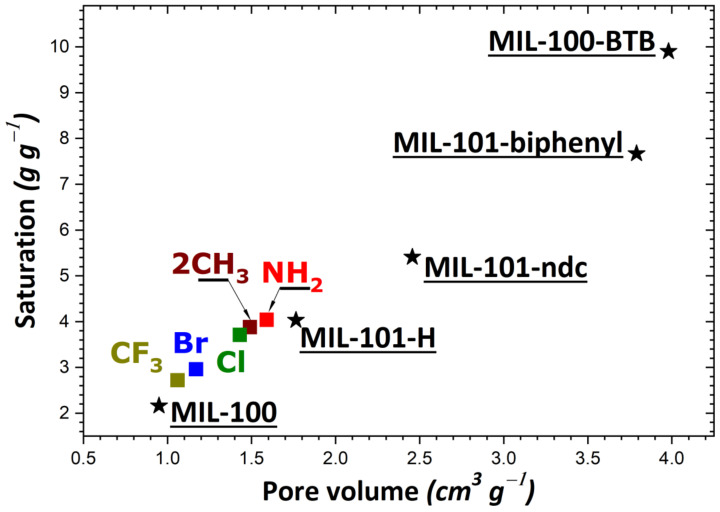
Evolution of the adsorption capacity of the adsorbents belonging to the MIL-100/MIL-101 series (MIL-100; MIL-101-ndc; MIL-101-biphenyl; MIL-100-BTB; MIL-101-X with X = H, NH_2_, 2CH_3_, Cl, Br, CF_3_) towards gaseous I_2_ at saturation as a function of pore volume.

To rationalize these trends, the impact of the theoretical specific surface area and pore volume (obtained following the procedure described in Section 2) was investigated as a result of structural functionalization. For the MIL-53 series, plots of adsorption capacity versus pore volume and specific surface area are given in Figure 2a,b, respectively. No correlation was established between saturation capacity and both textural parameters. On the contrary, clearer trends could be inferred from the analysis of the corresponding plots in Figure 3 for the UiO-66 series. It may be argued that the main difference between the two MOF series lies in the pore network: namely, linear pores in MIL-53 and interconnected cages in UiO-66. To provide further support for this hypothesis, some structures of the MIL-100 and MIL-101 series possessing interconnected cages were taken into consideration. A noticeable direct correlation was achieved this time, as can be seen in Figure 4.

Given the observed trends and correlations, it was possible to propose solid structures that presented high efficiency in adsorbing I_2_ from the gas phase. The members of the MIL-53 series were characterized by limited adsorption capacity, irrespective of the functional group grafted on the benzene ring. Contrary to this, the MIL-100 and MIL-101 series with large pores exhibited adsorption performances that were promising for efficient I_2_ capture.

For some selected MOFs with promising sorption performances, the impact of temperature was studied at 353 K (i.e., 80 °C) by determining enthalpy at low coverage and adsorption capacity at 1 bar. The resulting values are reported in Table 1 and compared with those obtained previously at 300 K.

A detailed analysis of the results reported in Table 1 reveals that adsorption enthalpy is only slightly modified by temperature, and values corresponding to 300 K and 353 K are fairly similar. In contrast, the amount of I_2_ adsorbed onto these porous solids is strongly dependent on temperature, especially in the case of mesoporous solids. For microporous solids (namely, the selected sampleswithin the UiO-66 and MIL-53 series), the amount adsorbed is relatively constant if one compares the two temperatures. Meanwhile, the amount of I_2_ uptaken by selected samples from the MIL-101/MIL-100 series is drastically modified as a function of temperature, and it strongly decreases when passing from 300 K to 353 K. From these results, it appears that the choice of the best performant samples may be also dependent on the operating conditions applied.

## 4. Conclusions

In conclusion, a research strategy based on a molecular simulation was tested to elucidate the effects of structural functionalization, the nature of pores, flexibility, and the presence of compensating cations on MOF retention capacity for I_2_ from the gas phase. This strategy aims to replace fastidious experiments while avoiding the potential health risks caused by radiation exposure. This study offers a rational approach to select the best adsorbents in view of the industrial uses envisaged here. These results may contribute to orienting choices of water-stable porous solids (within the framework of the present selection of materials) for applications involving the capture of either traces or large amounts of vapor (such as those released during nuclear accidents). As far as all adsorption properties are considered, MIL-100-BTB has been proven to exhibit the most promising performance at room temperature. MIL-100 possessing longer organic moieties and thus larger pores can even exceed the maximum adsorption capacity of the former sample and, as such, it may be used in the case of massive adsorption. In contrast, the capacity of MOF materials to retain traces of gaseous I_2_ on their surface argues in favor of MIL-101-2CH_3_, MIL-101-CF_3_, or UiO-66-CH_3_. They may be of greater interest in separation uses of porous materials subjected to the poisoning effect of water. In fact, they carry organic linkers that are generally not affected by the adsorption of water molecules. Calculations also show that the choice of solids is strongly dependent on the conditions of use of these solids. Indeed, mesoporous solids are greatly impacted by an increase in temperature, while microporous solids (UiO-66 and MIL-53 series) appear more stable. It follows that, at temperatures higher than room temperature, such solids could present an interesting alternative for I_2_ capture.

## Figures and Tables

**Table 1:** Comparison of theoretical values obtained from Monte Carlo simulations for enthalpy at low coverage (1 molecule per unit cell) and adsorption capacity at 1 bar as a function of temperature.

Sample	Enthalpy (kJ/mol)		I_2_ Amount Adsorbed at 1 bar (g/g)	
	300 K	353 K	300 K	353 K
**MIL-53-Br**	37.5	37.3	0.97	0.73
**UiO-66-CH_3_**	49.7	49.7	0.57	0.54
**UiO-66-2CH_3_**	53.7	53.6	0.60	0.45
**MIL-100-BTB**	37.2	36.7	2.30	0.80
**MIL-101-CF_3_**	52.2	51.9	1.68	0.50
**MIL-101-2CH_3_**	53.1	53.4	2.67	0.72
**MIL-101-biphenyl**	27.9	26.1	2.60	0.80

## Supplementary Materials

The following are available online at https://www.mdpi.com/article/10.3390/nano11092245/s1, Figure S1: Molecular conformations of the first I_2_ molecule adsorbed onto MOF structures belonging to the MIL-53 series functionalized by: (a) H, (b) CH_3_, (c) NH_2_, (d) Cl, (e) Br, as obtained based on the Monte Carlo simulations; Figure S2: Molecular conformations of the first I_2_ molecule adsorbed onto MOF structures belonging to the MIL-100/101 series: (a) MIL-100, (b) MIL-101-Cl, (c) MIL-101-Br, (d) MIL-101-2CF_3_, (e) MIL-101-NH_2_, (f) MIL-101-biphenyl, (g) MIL-100-BTB, as obtained based on the Monte Carlo simulations; Figure S3: Molecular conformations of the first I_2_ molecule adsorbed onto MOF structures belonging to the UiO-66 series: (a) H, (b) Cl, (c) Br, (d) NH_2_, (e) NO_2_, (f) CH_3_, (g) 2CH_3_, as obtained based on the Monte Carlo simulations; Figure S4: Adsorption isotherms for I_2_ onto MOF structures belonging to the MIL-53 series, as calculated based on the Monte Carlo simulations; Figure S5: Adsorption isotherms for I_2_ onto MOF structures belonging to the UiO-66 series, as calculated based on the Monte Carlo simulations; Figure S6: Adsorption isotherms for I_2_ onto MOF structures belonging to the MIL-101 series, as calculated based on the Monte Carlo simulations; Figure S7: Adsorption isotherms for I_2_ onto MOF structures belonging to the modified MIL-100/MIL-101 solids, as calculated based on the Monte Carlo simulations; Figure S8: Adsorption isotherms for I_2_ onto MOF structures belonging to the cation-saturated Zn-BTeC family, as calculated based on the Monte Carlo simulations; Figure S9: Adsorption isotherms for I_2_ onto MOF structures belonging to the anion-saturated MIL-127, as calculated based on the Monte Carlo simulations.
