# Supplementary material for: Impact of Structural Functionalization, Pore Size, and Presence of Extra-Framework Ions on the Capture of Gaseous I2 by MOF Materials
Source: Nanomaterials (Basel). 2021 Aug 30;11(9):2245. doi: 10.3390/nano11092245 (PMC8467223; doi:10.3390/nano11092245)
Supplement: Supplementary file 1 [file nanomaterials-11-02245-s001.zip › nanomaterials-1364760-supplementary.pdf]

Supplementary Material

Impact of Structural Functionalization, Pore Size, and Presence of Extra-Framework Ions on the Capture of Gaseous I_2 by MOF Materials

Fabrice Salles * and Jerzy Zajac

ICGM, Université Montpellier CNRS ENSCM, 34090 Montpellier, France; jerzy.zajac@umontpellier.fr
* Correspondence: fabrice.salles@umontpellier.fr

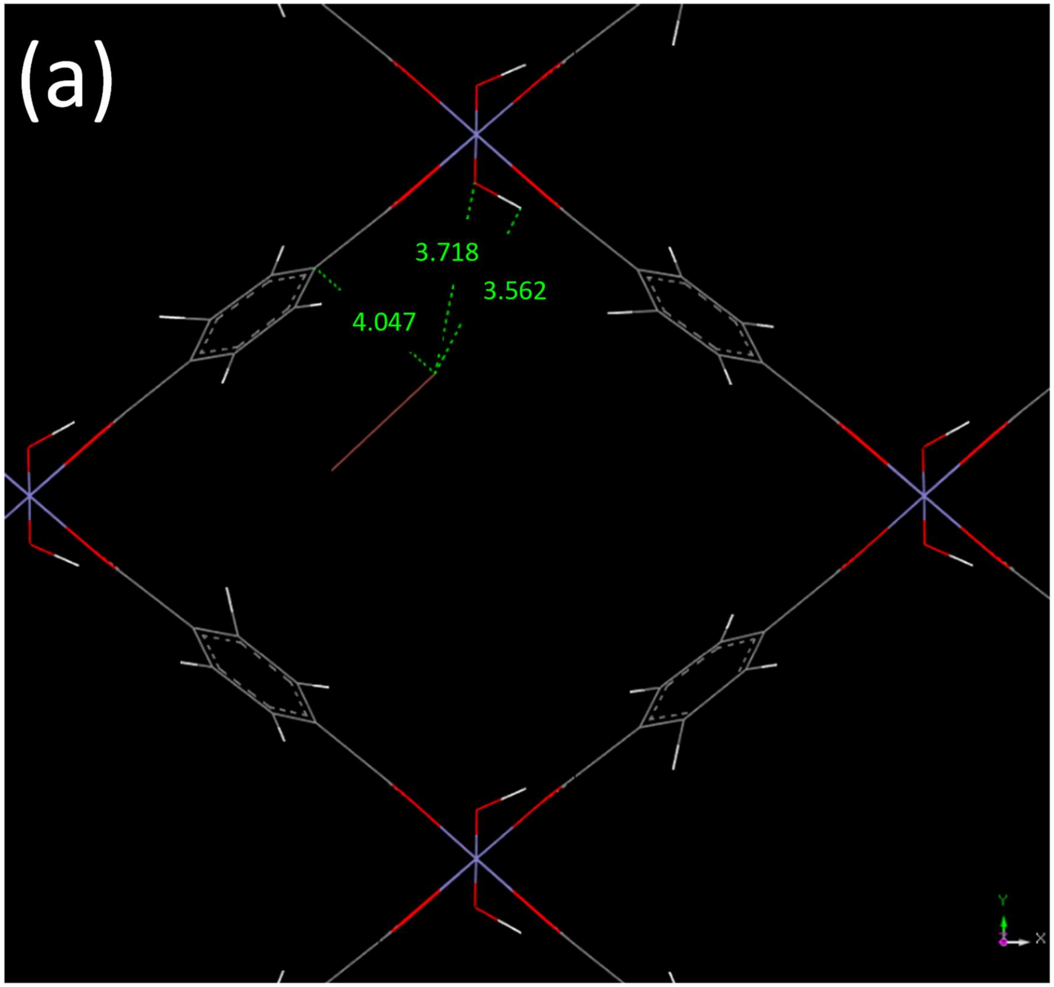

(a)
3.718
3.562
4.047
Y
X

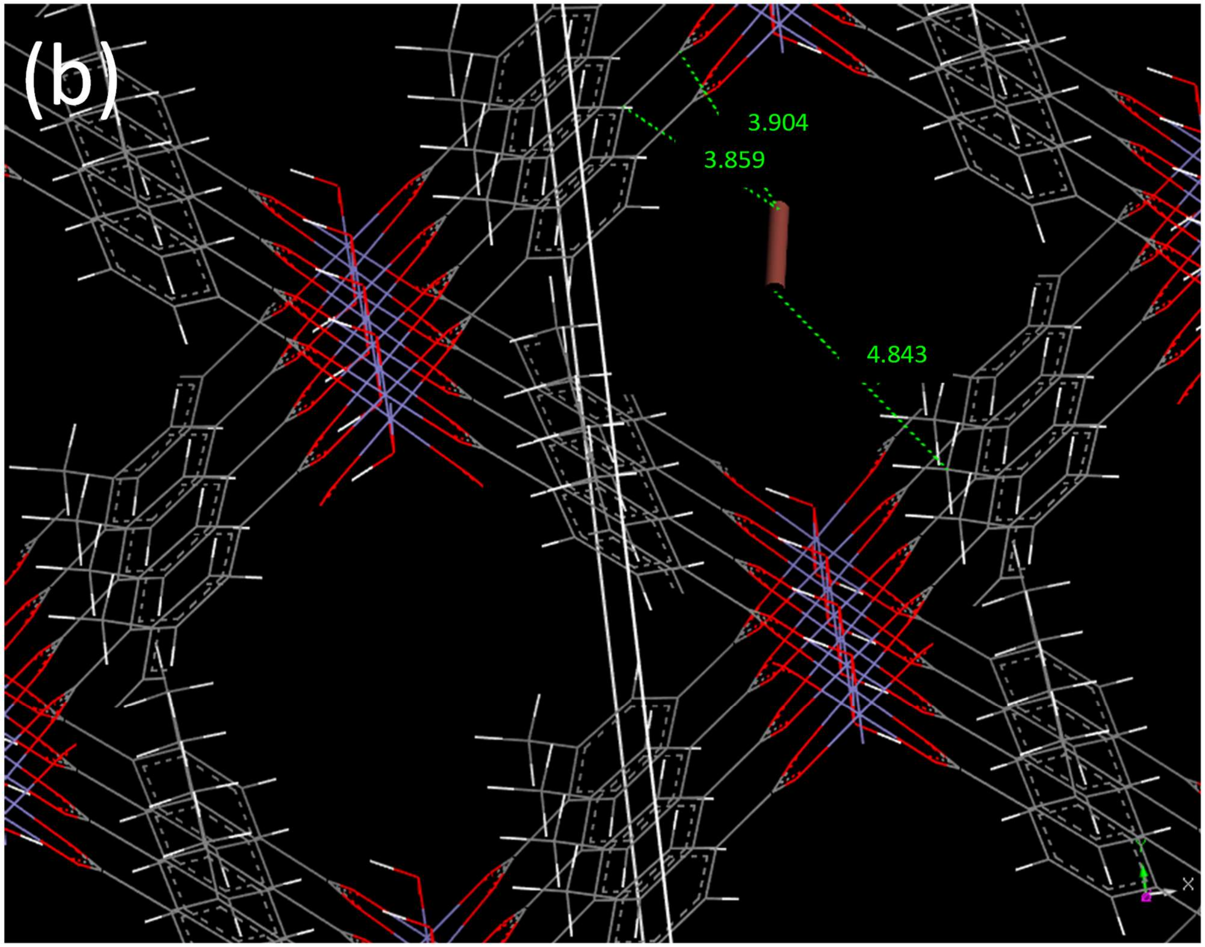
(b)
3.904
3.859
4.843
Y
X

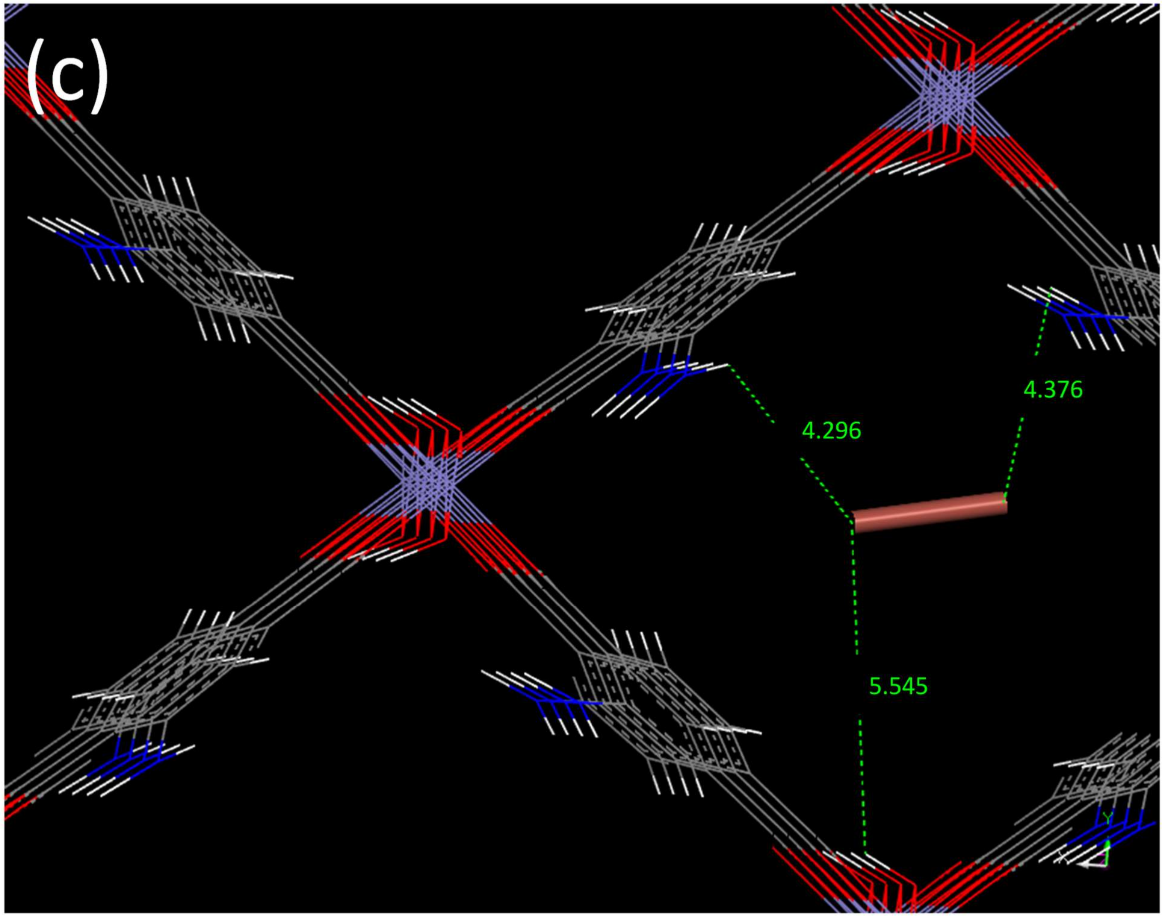
(c)
4.296
4.376
5.545

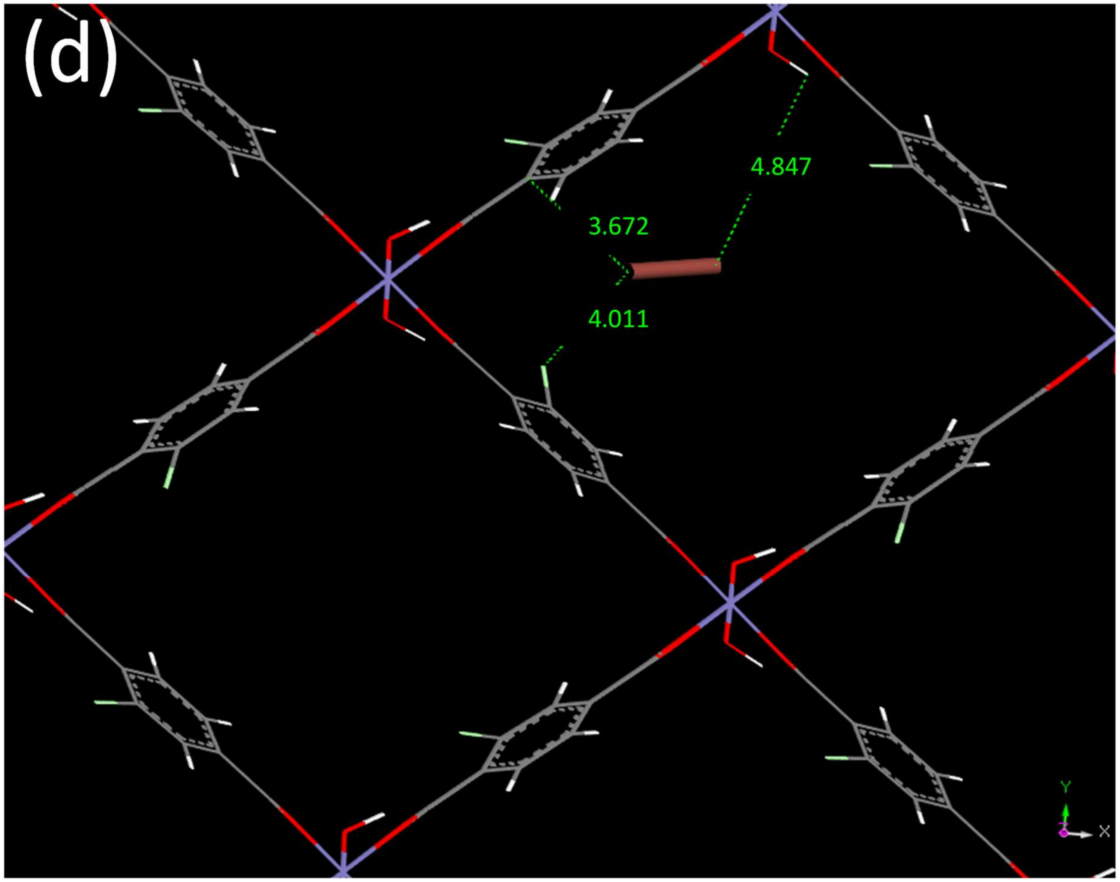
(d)
4.847
3.672
4.011
Y
X

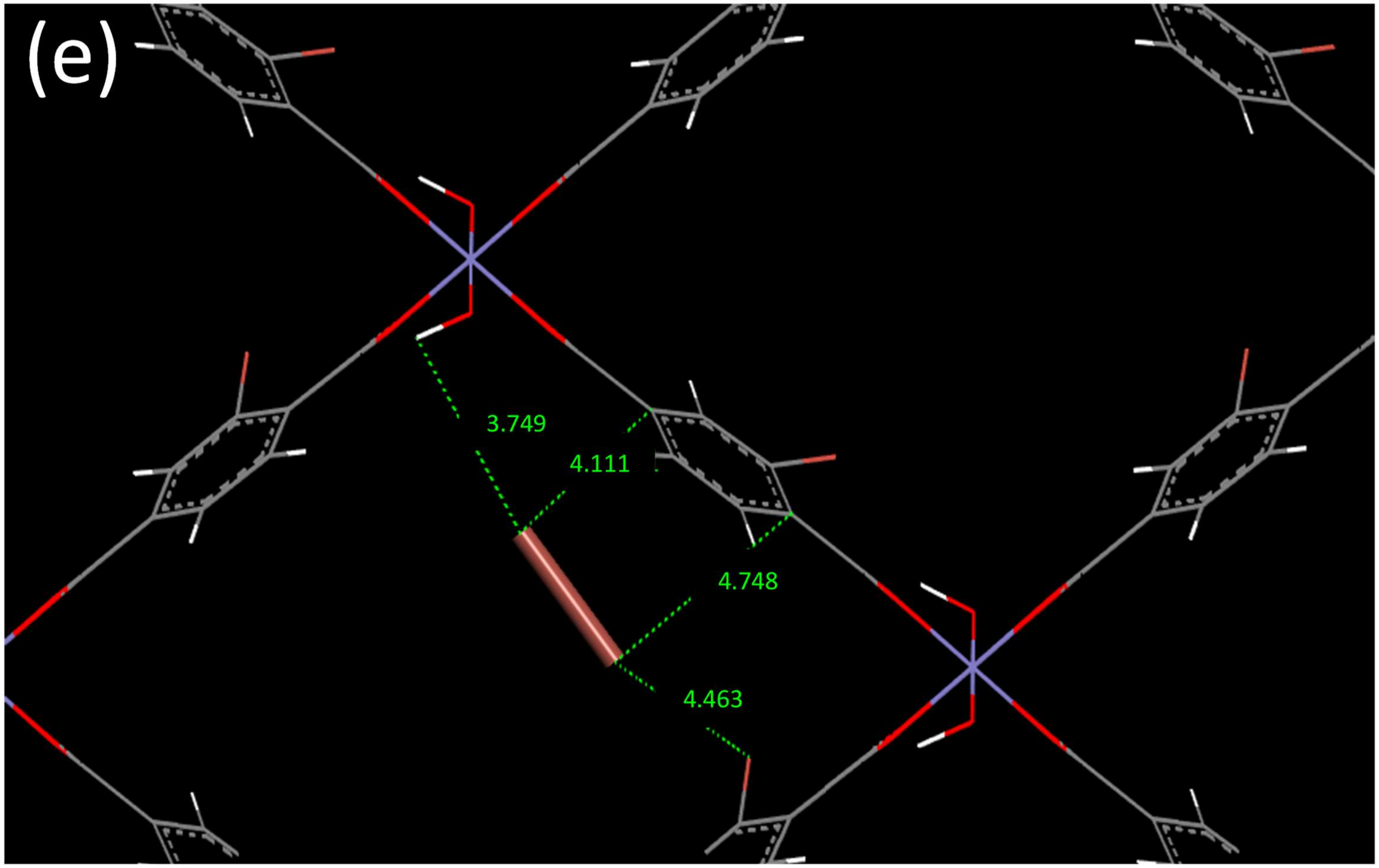

Figure S1. Molecular conformations of the first I_2 molecule adsorbed onto MOF structures belonging to the MIL-53 series functionalized by: (**a**) H, (**b**) CH_3, (**c**) NH_2, (**d**) Cl, (**e**) Br, as obtained based on the Monte Carlo simulations.

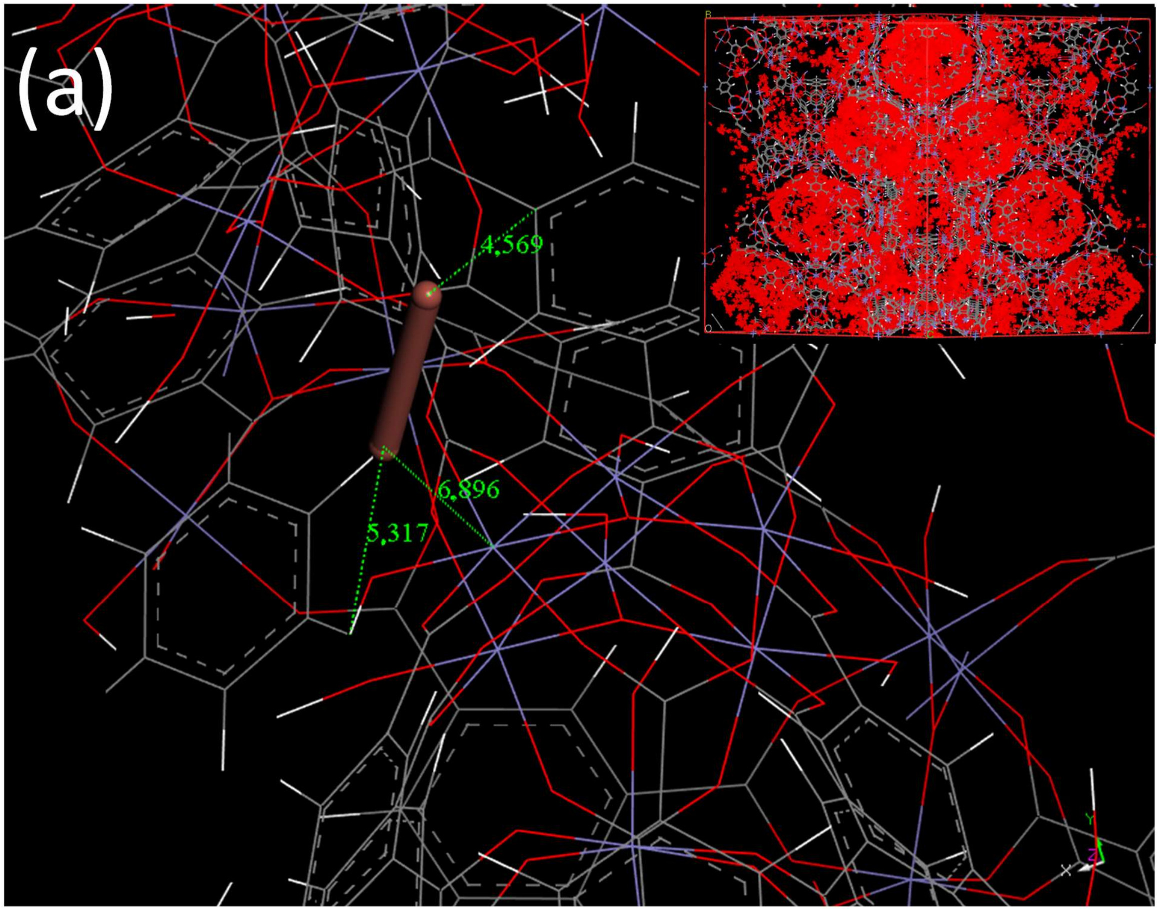

(a)
4.569
6.896
5.317

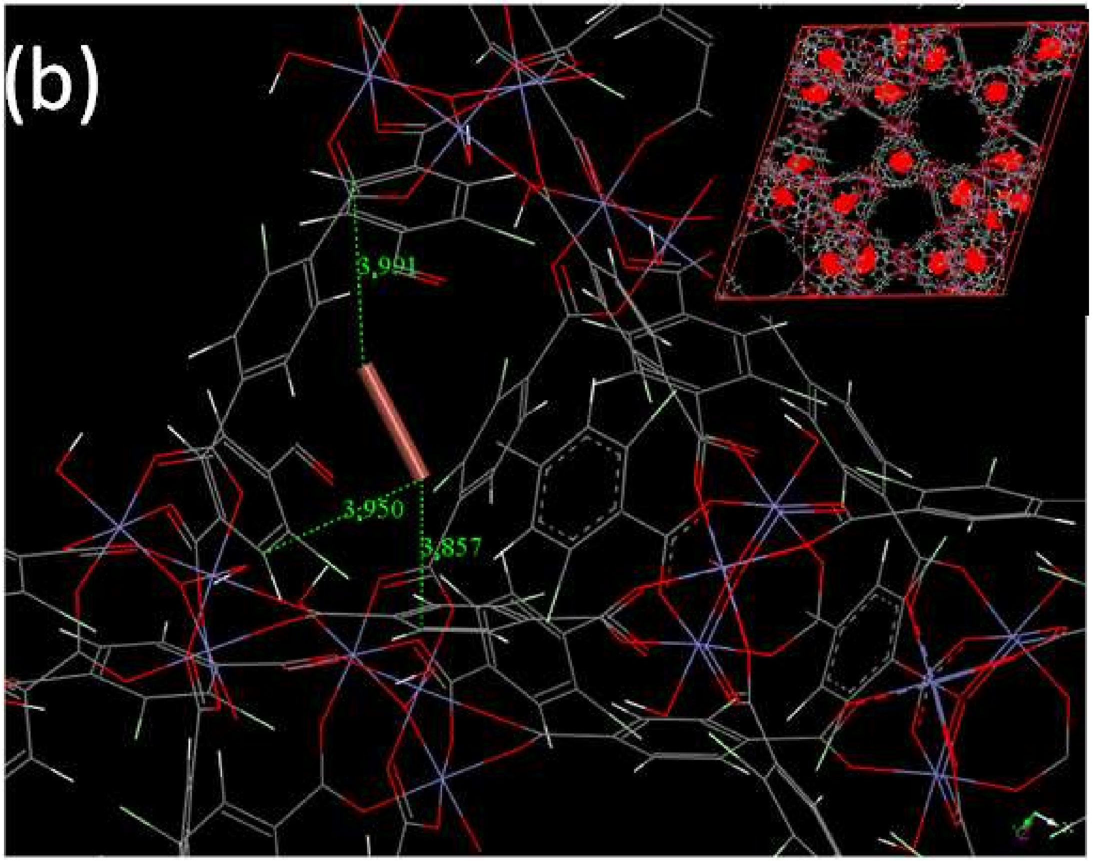
(b)
3.991
3.950
3.857

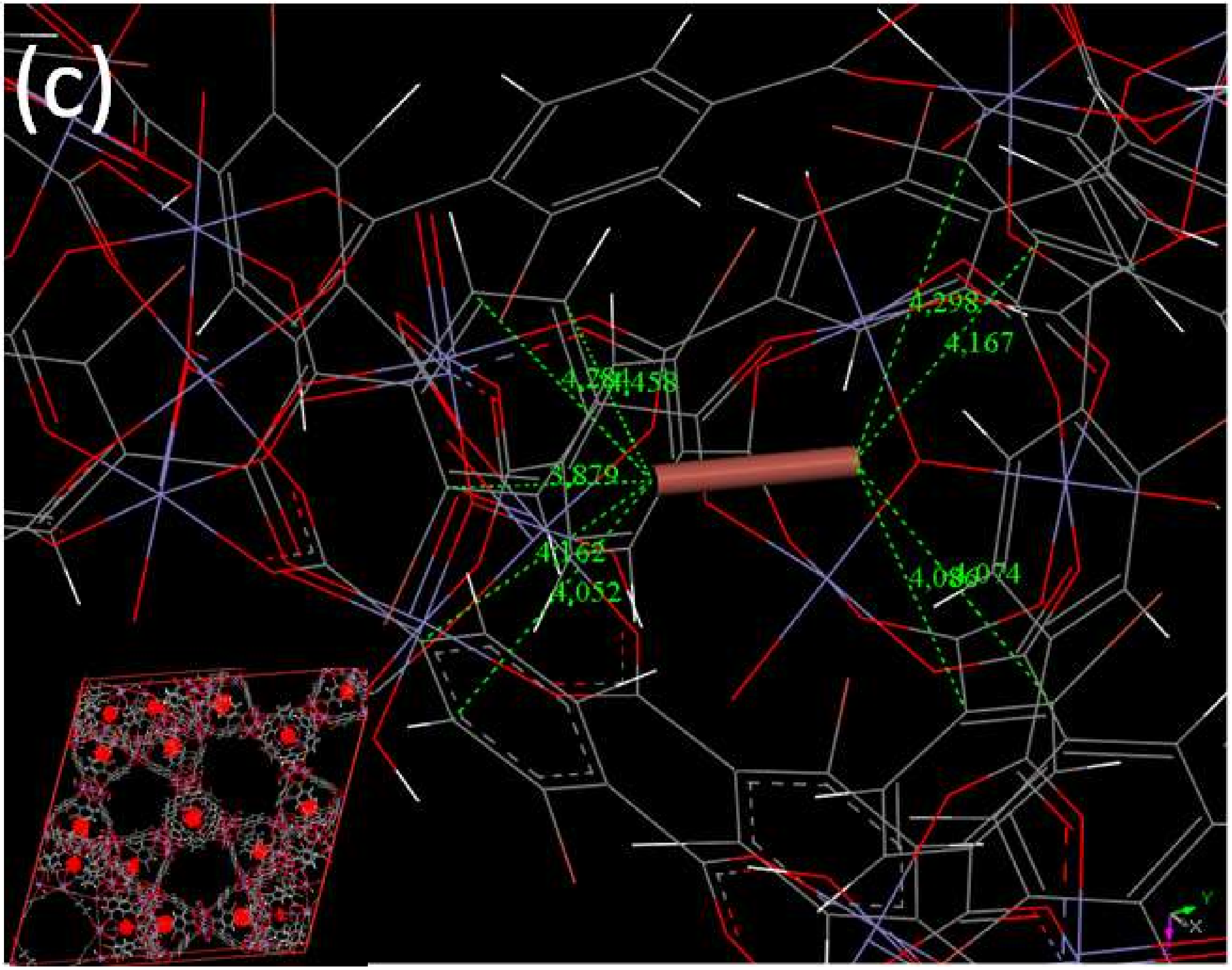

(c)
4,298
4,167
3,879
4,162
4,052

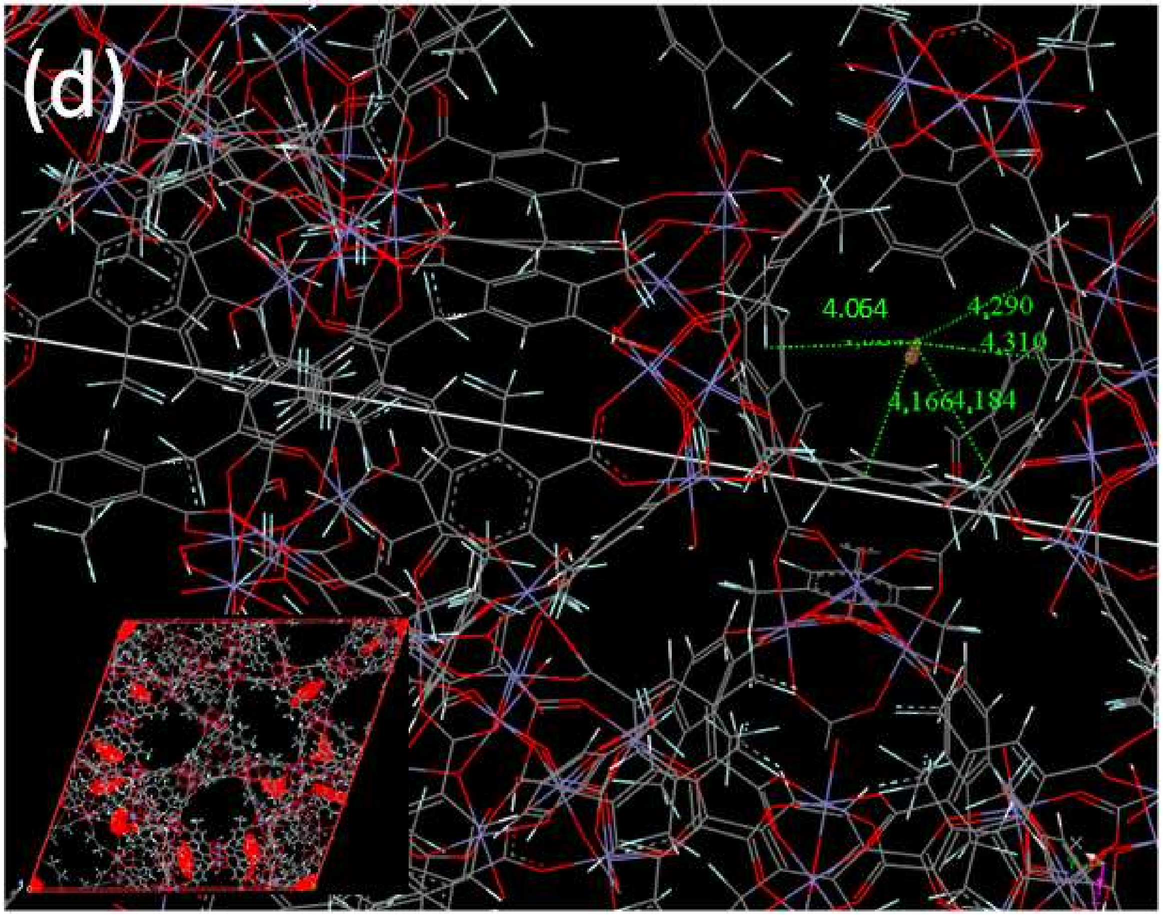
(d)
4.064
4.290
4.310
4.1664.184

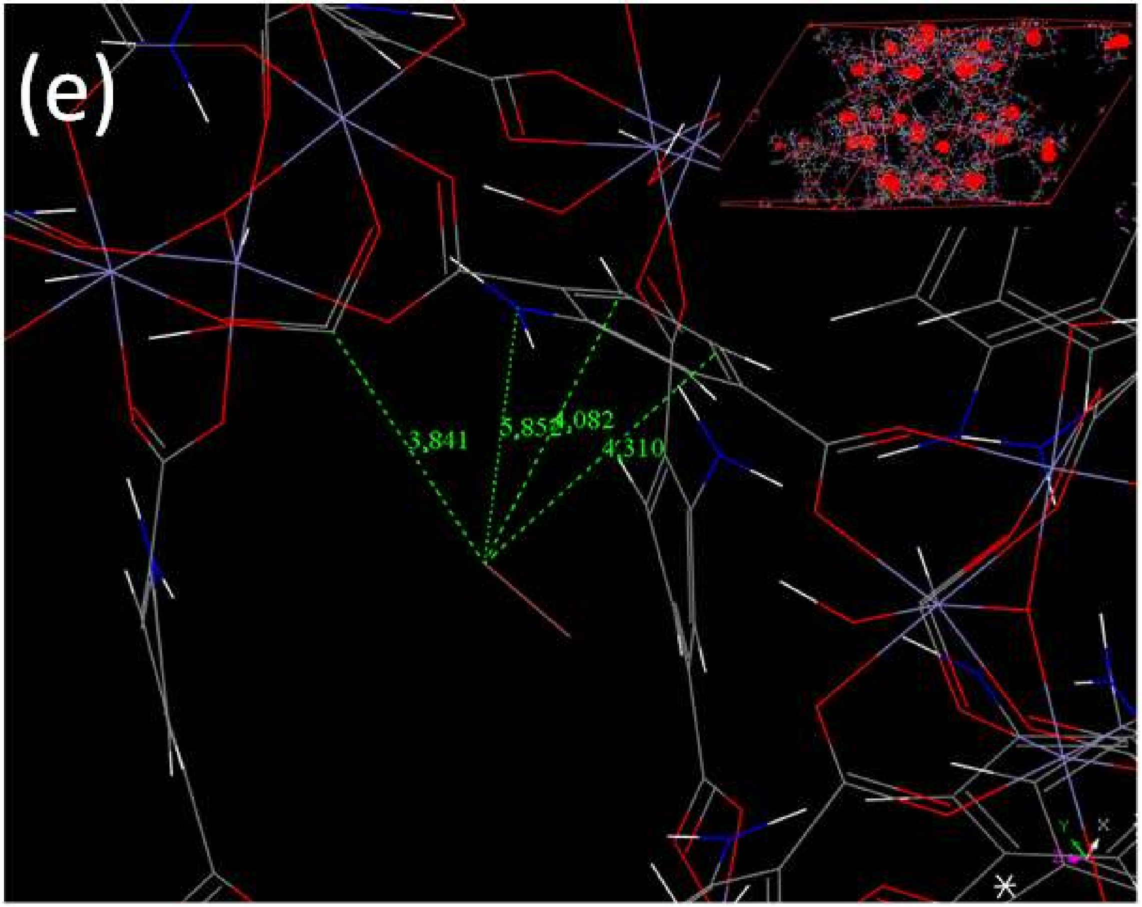

(e)
3.841
5.852
4.082
4.310

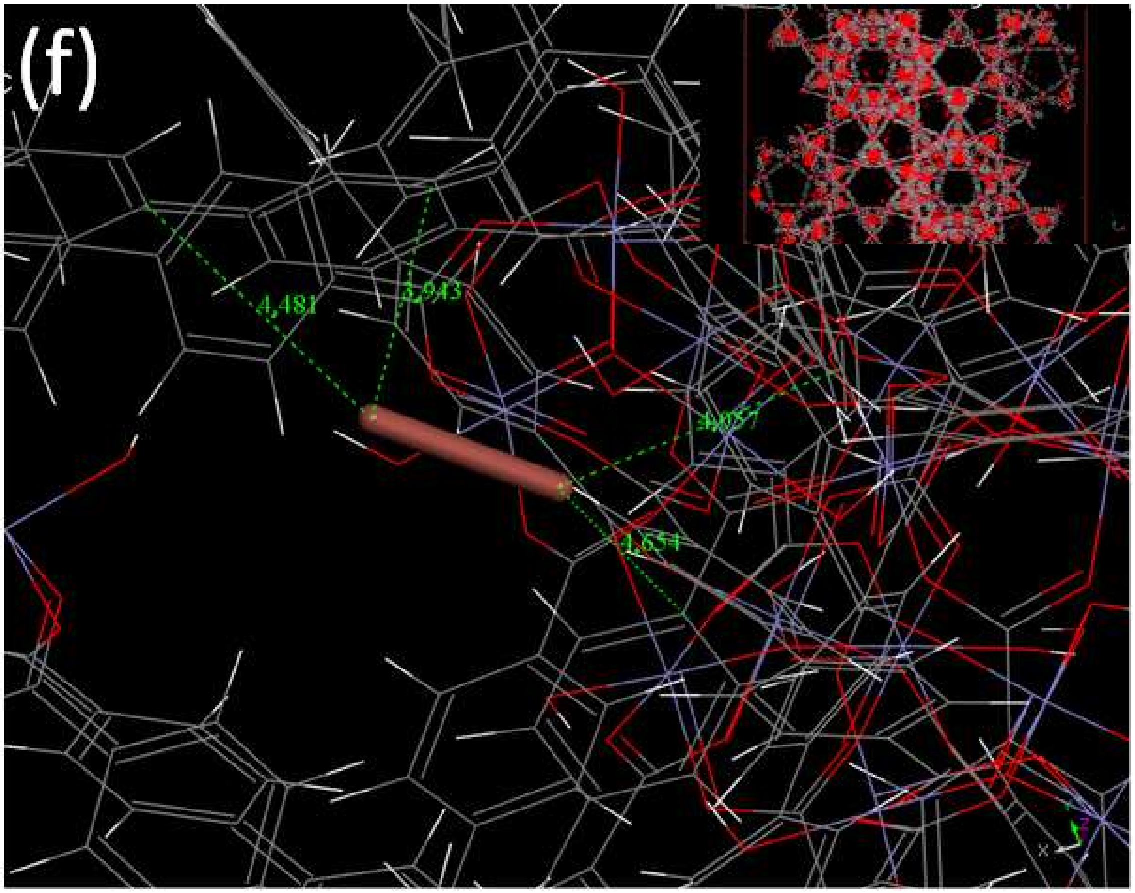
(f)
4.481

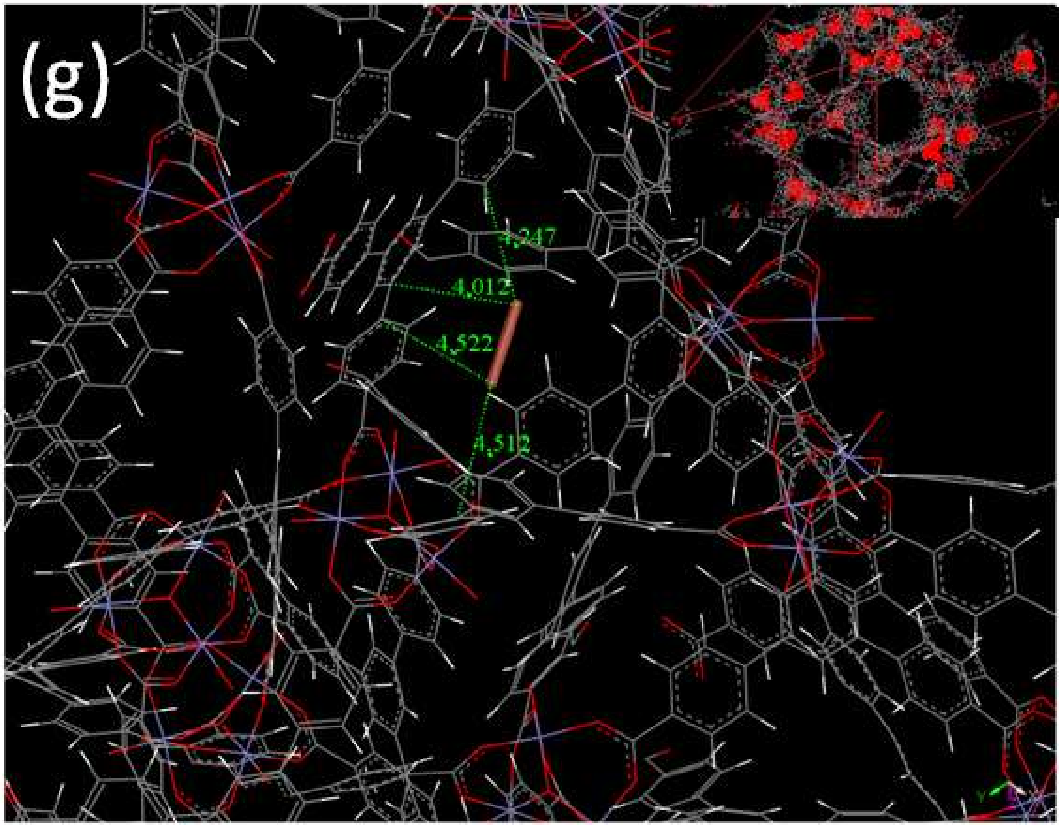

Figure S2. Molecular conformations of the first I_2 molecule adsorbed onto MOF structures belonging to the MIL-100/101 series: (**a**) MIL-100, (**b**) MIL-101-Cl, (**c**) MIL-101-Br, (**d**) MIL-101-2CF_3, (**e**) MIL-101-NH_2, (**f**) MIL-101-biphenyl, (**g**) MIL-100-BTB, as obtained based on the Monte Carlo simulations. At the corner of the picture, the density plot (red points) to localize the main adsorption sites is given for each sample.

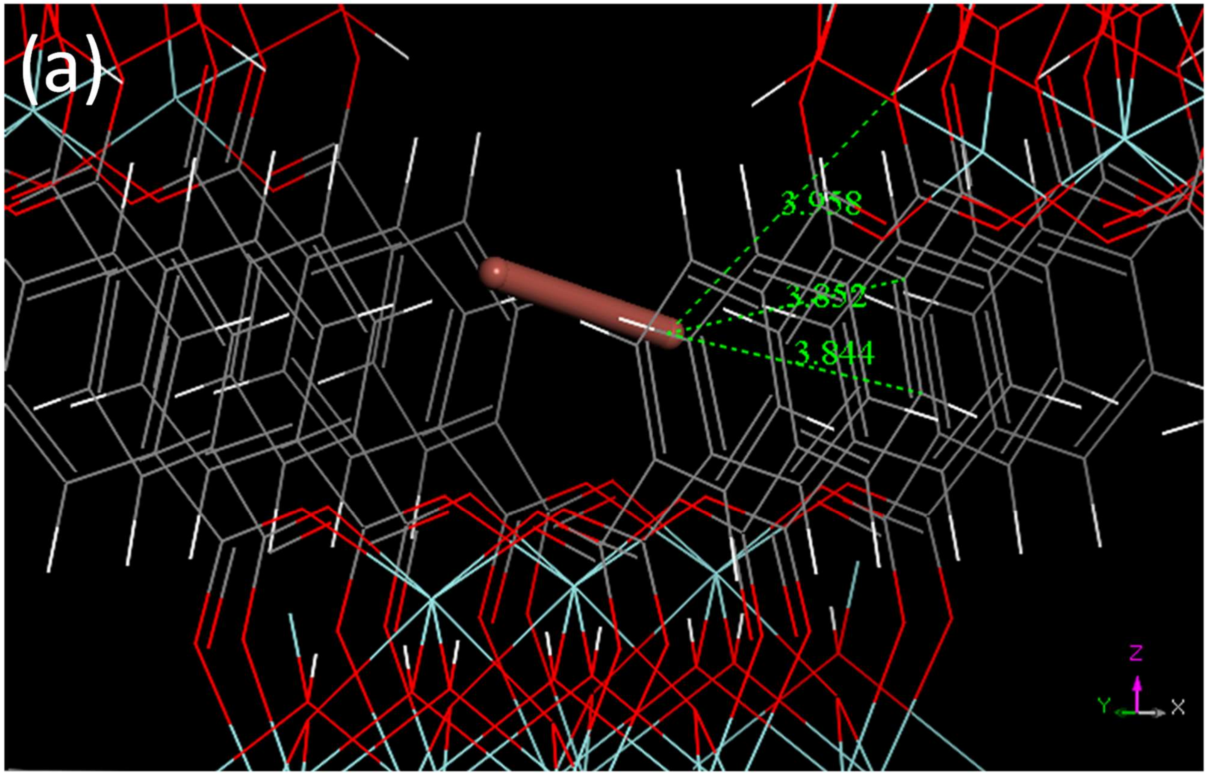

(a)
3.958
3.852
3.844
Z
Y
X

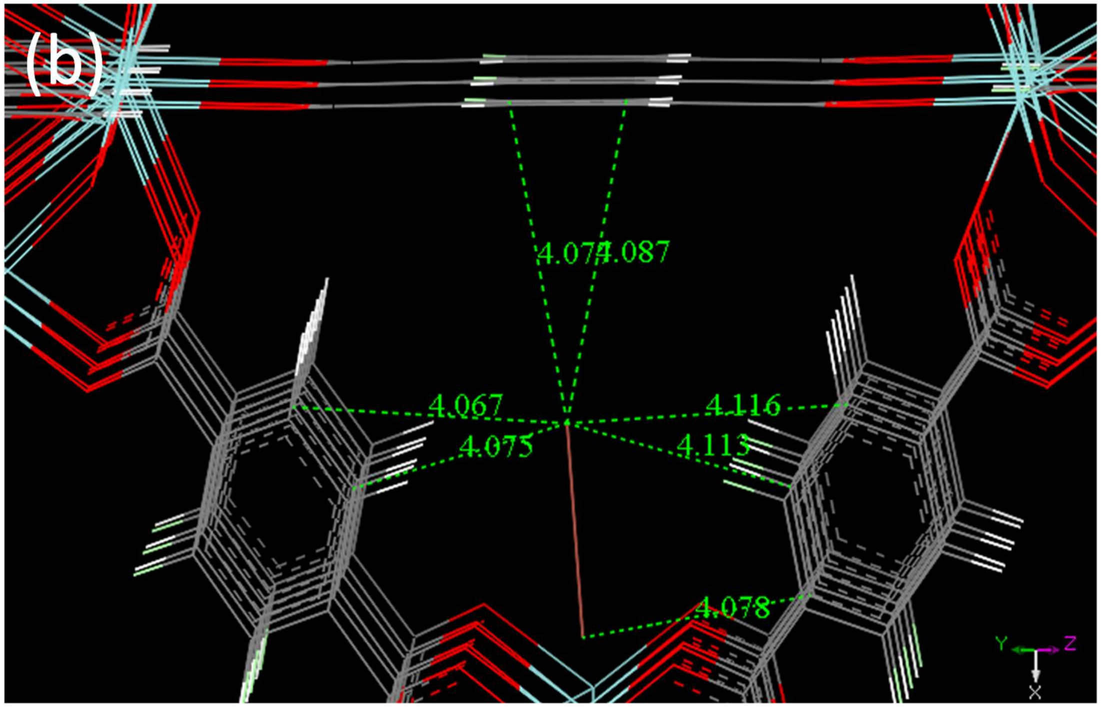
(b)
4.074
4.087
4.067
4.075
4.116
4.113
4.078
Y
Z
X

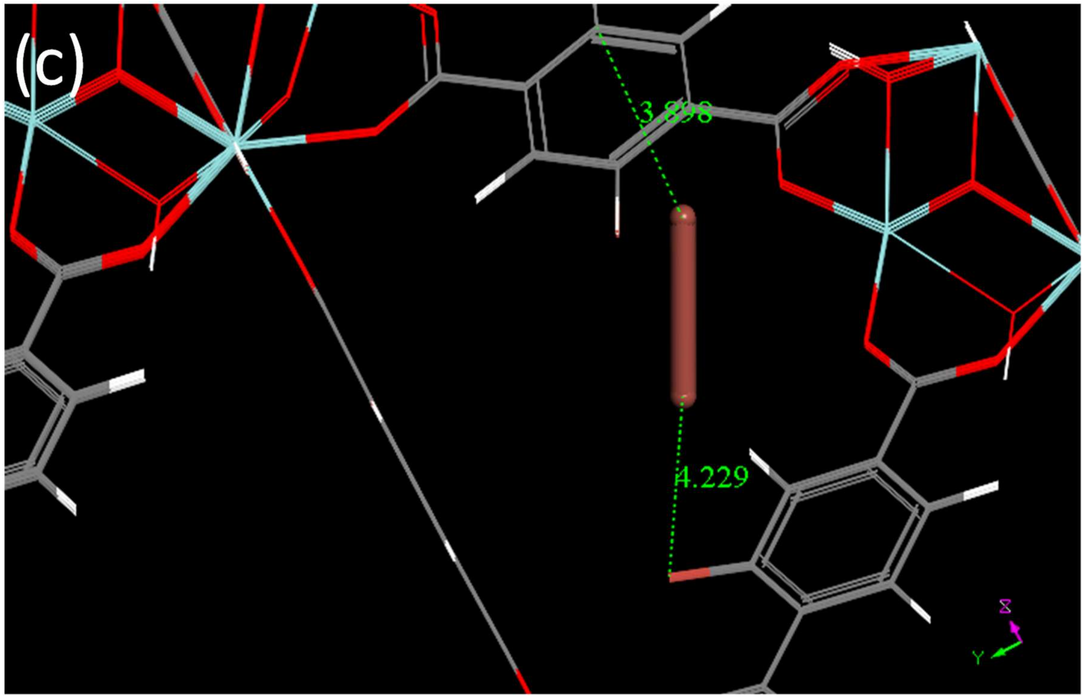

(c)
3.898
4.229
Z
Y

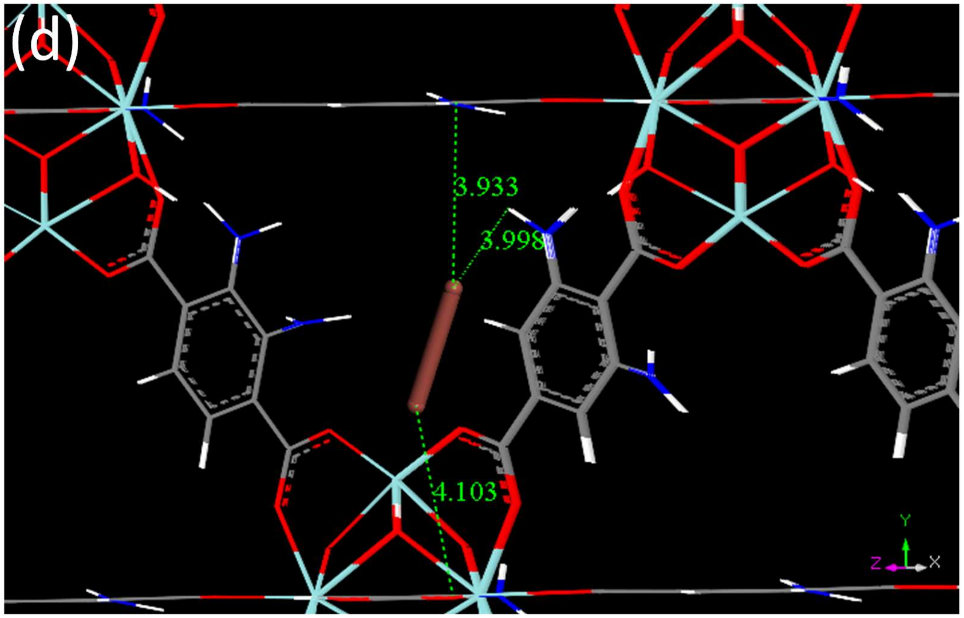
(d)
3.933
3.998
4.103
Y
Z
X

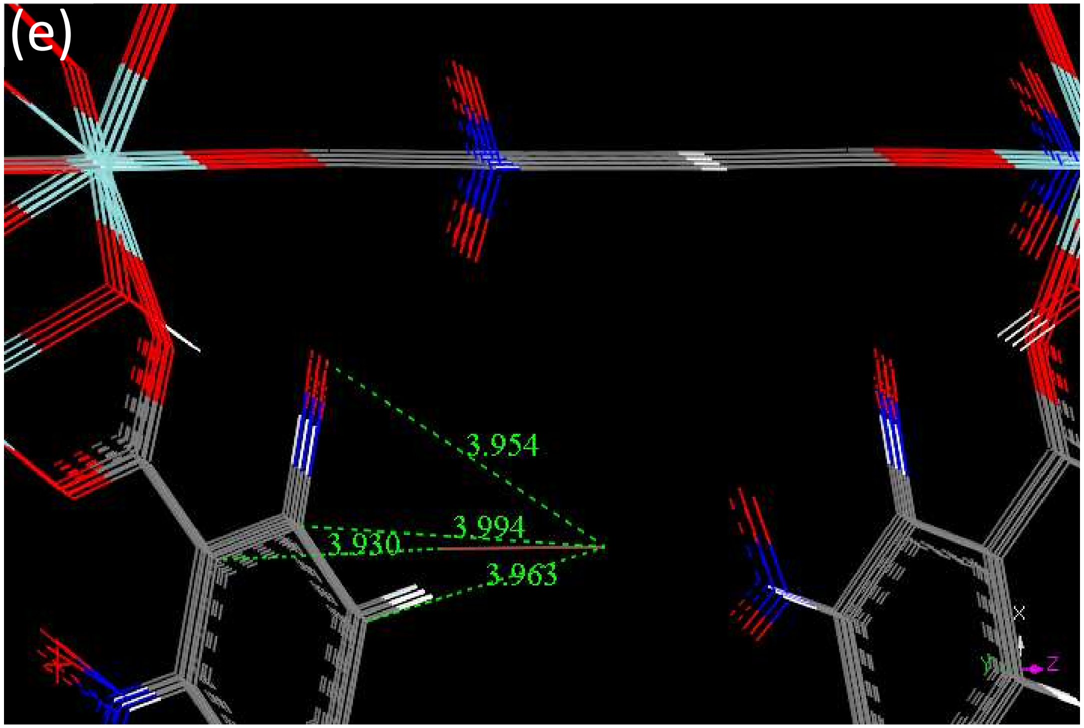
(e)
3.954
3.994
3.930
3.963
X
Y
Z

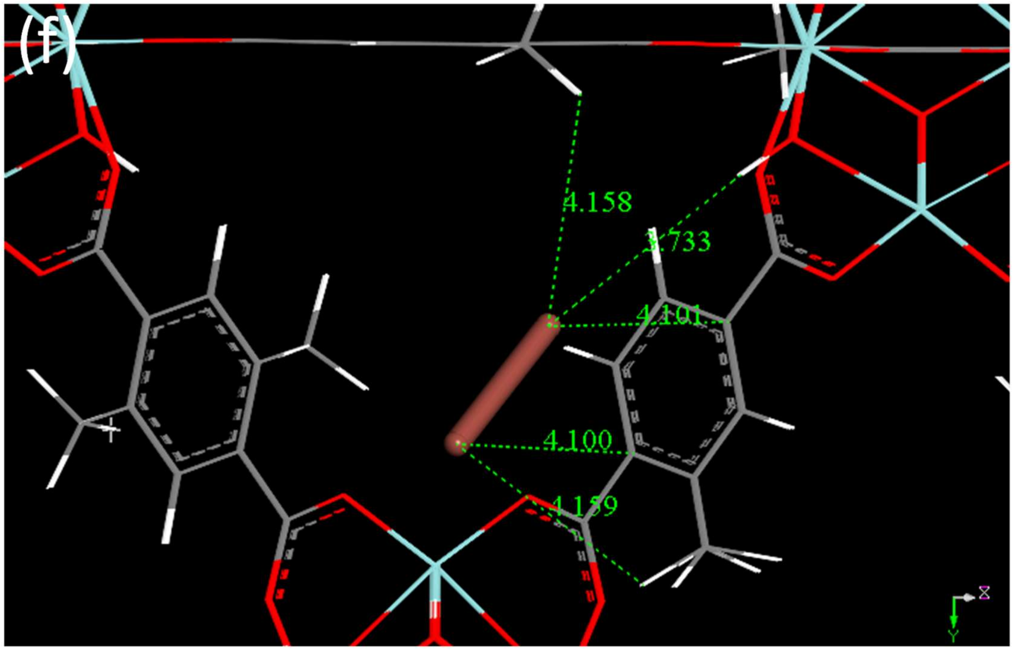

(f)
4.158
3.733
4.101
4.100
4.159
Y

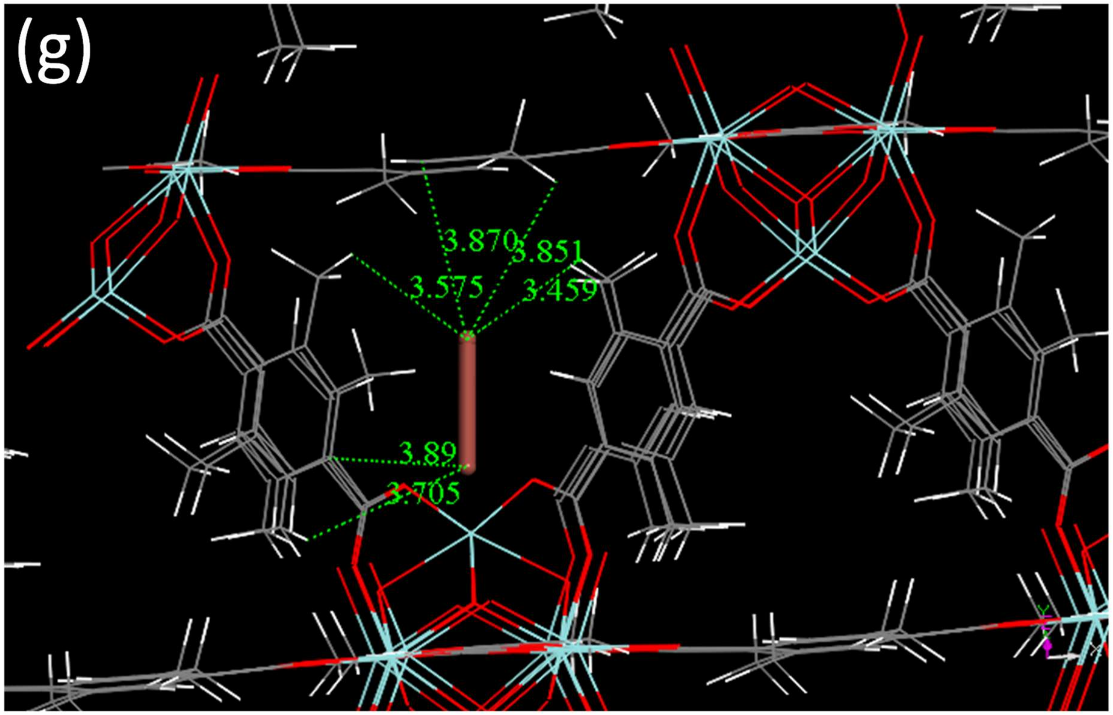

Figure S3. Molecular conformations of the first I_2 molecule adsorbed onto MOF structures belonging to the UiO-66 series: (**a**) H, (**b**) Cl, (**c**) Br, (**d**) NH_2, (**e**) NO_2, (**f**) CH_3, (**g**) $2CH_3$, as obtained based on the Monte Carlo simulations

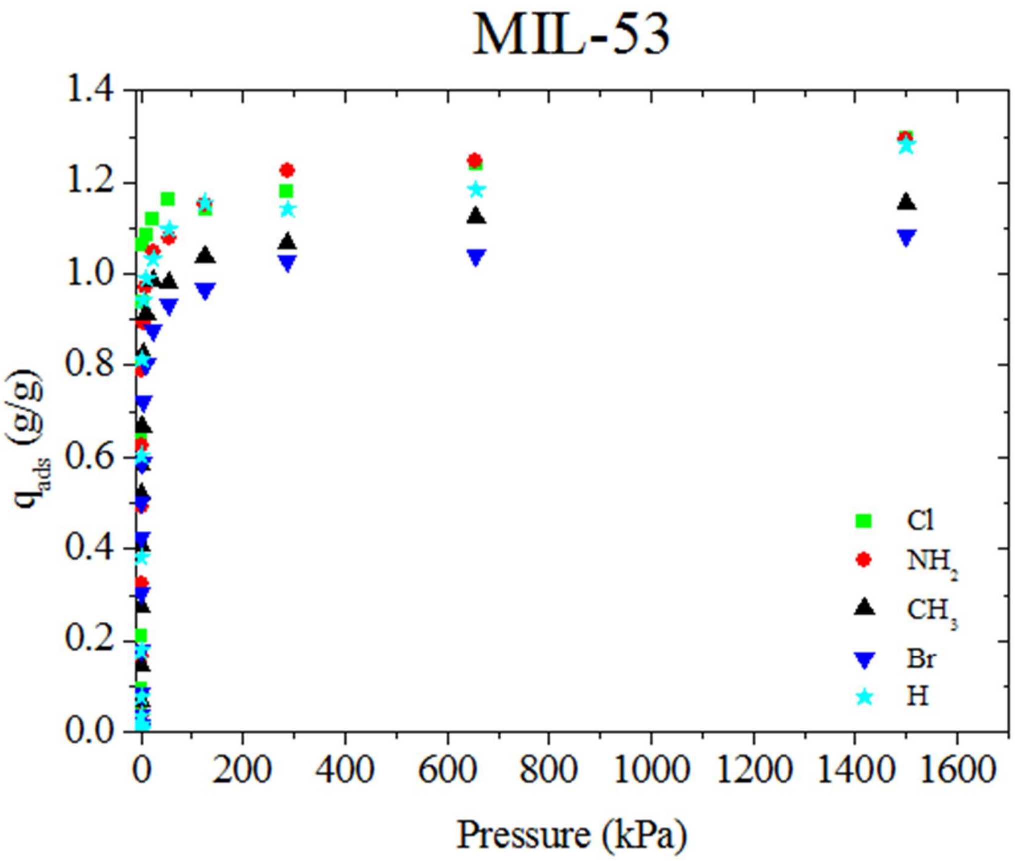

Figure S4. Adsorption isotherms for I_2 onto MOF structures belonging to the MIL-53 series, as calculated based on the Monte Carlo simulations

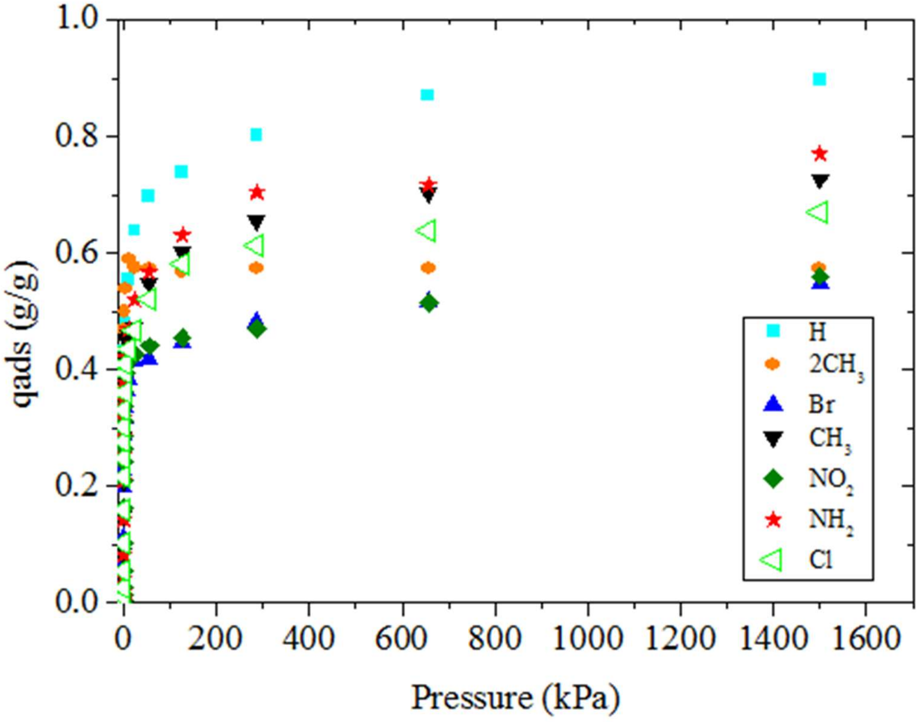

Figure S5. Adsorption isotherms for I_2 onto MOF structures belonging to the UiO-66 series, as calculated based on the Monte Carlo simulations

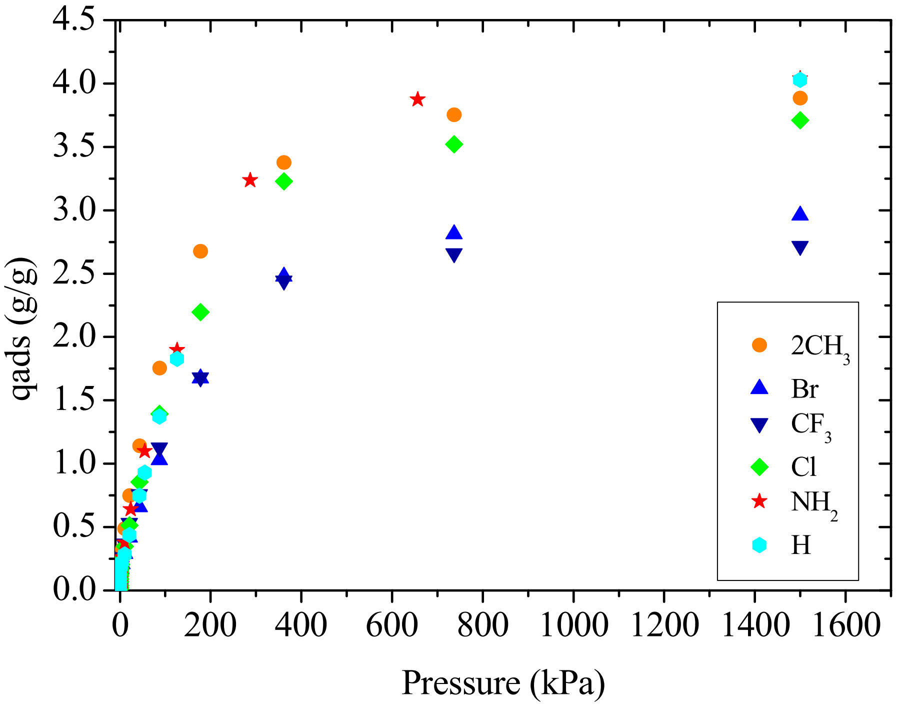

Figure S6. Adsorption isotherms for I_2 onto MOF structures belonging to the MIL-101 series, as calculated based on the Monte Carlo simulations

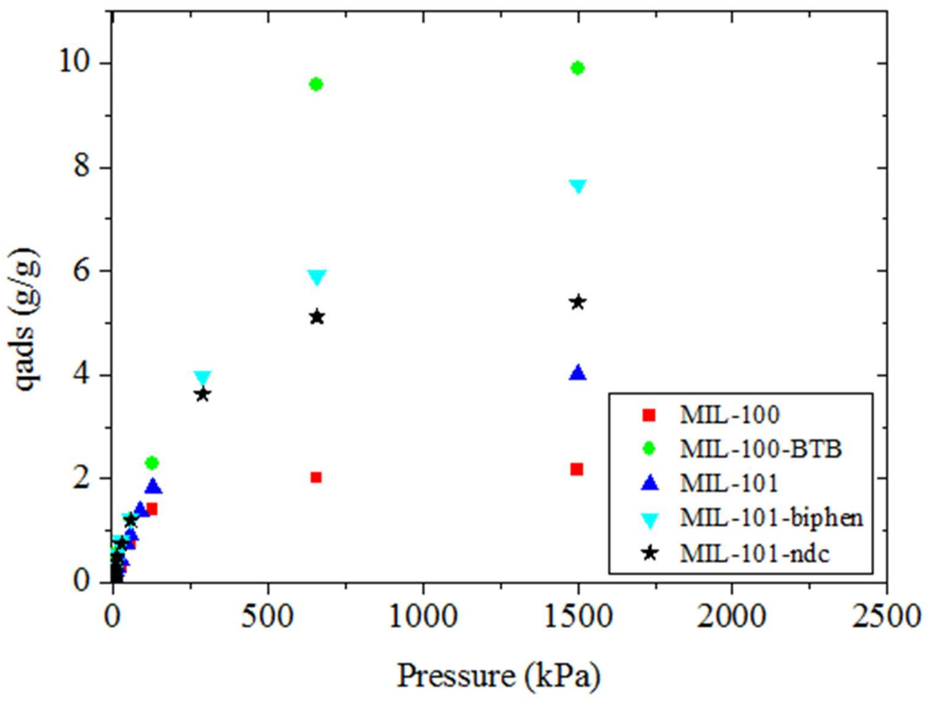

Figure S7. Adsorption isotherms for I_2 onto MOF structures belonging to the modified MIL-100/MIL-101 solids, as calculated based on the Monte Carlo simulations.

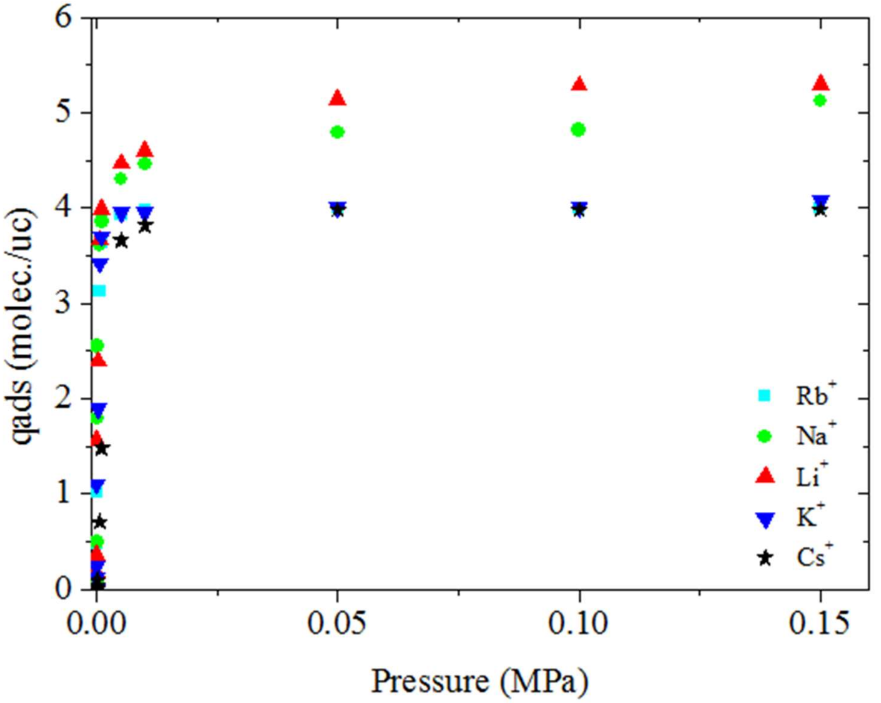

Figure S8. Adsorption isotherms for I_2 onto MOF structures belonging to the cation-saturated Zn-BTeC family, as calculated based on the Monte Carlo simulations.

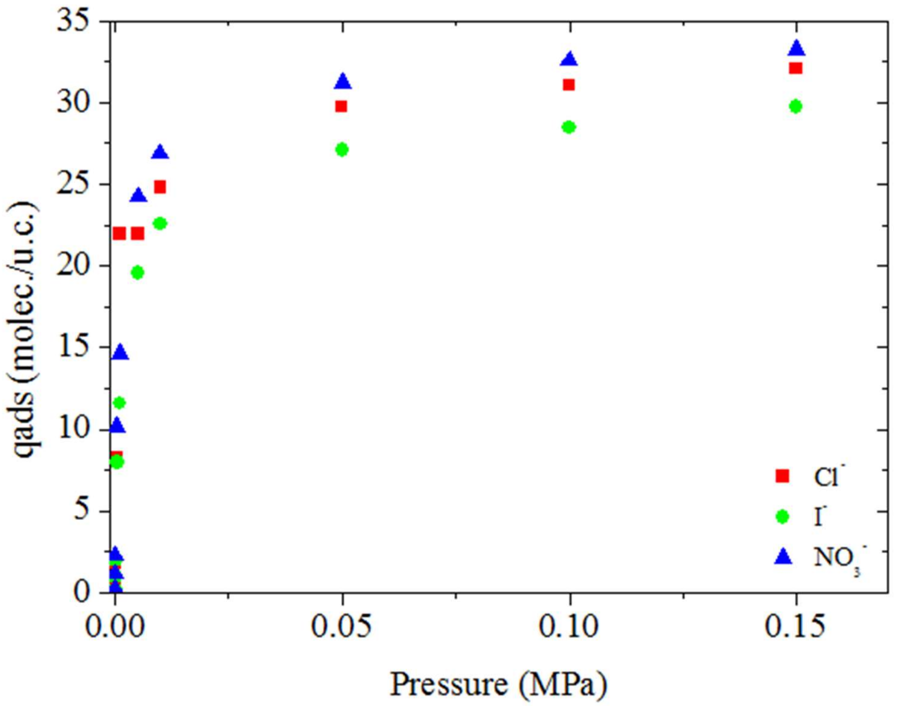

Figure S9. Adsorption isotherms for I_2 onto MOF structures belonging to the anion-saturated MIL-127, as calculated based on the Monte Carlo simulations.
